# Interlaminar Shear Strength Change and Storage Life Prediction of Carbon Fiber/Epoxy Composites with Hygrothermal Accelerated Aging

**DOI:** 10.3390/polym16081109

**Published:** 2024-04-16

**Authors:** Jinjuan Fan, Qin Zhang, Xinwen Chen, Yuhuai He

**Affiliations:** 1 AECC Beijing Institute of Aeronautical Materials, Beijing 100095, China; 2 Beijing Key Laboratory of Aeronautical Materials Testing and Evaluation, Beijing 100095, China

**Keywords:** carbon fiber-reinforced resin-based composite, hygrothermal accelerated aging, interlaminar shear strength, failure mechanism, storage life prediction

## Abstract

In order to investigate the durability of fiber-reinforced polymer composites in hygrothermal environments, hygrothermal accelerate aging tests, for 360 days at 70 °C, RH70%; 70 °C, RH85%; 85 °C, RH70%; and 85 °C, RH85% and natural storage for 2 years in Guangzhou, China, were carried out for composite laminates. Then, the moisture absorption and interlaminar shear strength were measured. The hygrothermal damage mechanism of the composite was studied by Fourier transform infrared (FTIR), differential scanning calorimetry (DSC), thermogravimetric analysis (TGA), and field emission scanning electron microscopy (FSEM). A dual stress storage life prediction model and the equivalent relationship between natural storage and hygrothermal acceleration were established. The results show that the order of moisture absorption rates, moisture absorption contents, and the severity effect order on the interlaminar shear strength is RH85%; 85 °C > 70 °C; RH85% > 85 °C; RH70% > 70 °C; and RH70%. The time to achieve an effective moisture absorption balance is opposite to this. The moisture absorption rate meets Fick’s law before the effective moisture absorption balance, and then shows a linear trend. The interlayer shear strength still decreases exponentially with aging, which is mainly caused by the resin plasticization and interface weakening. Hygrothermal accelerated aging for 13.4831 days at 85 °C; RH85% is equivalent to that for one-year actual storage in Guangzhou. According to the failure criterion of shear strength decreasing to 77%, the storage life of T700/epoxy in Guangzhou is 14.4661 years.

## 1. Introduction

Carbon fiber-reinforced polymer composites have been widely used in the aerospace field because of their advantages of high specific strength and specific modulus, good designability, good fatigue resistance, and easy large-scale integral molding. The main reinforcing material currently used in polymer composites is T300. T700 and T800 carbon fibers with high strength and high toughness are the new generation of reinforcing materials [1,2].

According to the requirements of aerospace system applications, composite structural components should be suitable for long-term storage and ready for use at any time. Thus, polymer composite structures are inevitably exposed to environments with, for example, UV, high temperatures, high humidity, and high salt spray, which would lead to material changes, reduce their performance, and affect their durability [3,4,5]. Wang [6] studied the simulated marine environment aging behavior of a T300-reinforced polymer composite and found that the deterioration severity order of aging methods is hydrothermal aging, salt spray, and salt water immersion. Most research indicates that hygrothermal is one of the main environmental factors leading to performance decrease in polymer composites [6,7,8,9,10].

The effects of a hygrothermal environment on polymer composites were investigated by accelerated testing due to the fact that natural aging tests would take a long time [11,12,13,14,15]. The accelerated testing method subjects the specimen to a controlled and simpler, but more severe, test condition than the service environment and accelerates the material property degradation. Simultaneously, environmental effect factors can be separated into multiple influencing factors such as temperature, humidity, ultraviolet, salt mist, etc. Single-factor acceleration tests or coupled-factor acceleration tests can be conducted to obtain the key environmental factors that affect material performance. This method, combined with predictive models, can effectively save experimental time and costs.

Yang [16], Peret [17], Almeida [18], Sui [19], and Kesentini [20] found that the moisture absorption characteristics of carbon fiber-reinforced polymer composites, under water immersion at temperatures of 25 °C, 70 °C, and 85 °C and hygrothermal environments of 80 °C, RH85%, and 80 °C, RH 90%, conformed to Fick’s law. Wu Rui [21] found that the later moisture absorption behavior of polymer composites deviated from Fick’s law. Wang et al. [6,16,17] found that the tensile performance was less affected, the interlayer shear performance decreased rapidly, and the resin matrix underwent aging degradation in hygrothermal environments. Niu Yifan et al. [22,23] found that the swelling and plasticization of the resin after moisture absorption led to a significant decrease in the shear strength, and that a brittle fracture formed by the coupling of shear and compression; the interface debonding and resin hydrolysis led to a decrease in bending strength, and the fracture form was compressive brittle fracture. Liu [24] analyzed the failure mechanism of resin-based composite materials in hygrothermal environments, and found that the molecular chain structure of the resin would change after moisture absorption. DSC testing showed the glass transition temperature decrease in the resin. 

Usually, shear and compression properties, which were significantly affected by the hygrothermal environment, were used as life-characterizing parameters. The degradation of physical and chemical properties is also used for life prediction; for example, Wani [25] used dynamic mechanical analysis to predict storage life prediction. There are two main types of life prediction models: one is a dynamic model or empirical model with a certain physical meaning, including the single factorial Arrhenius Model and Eyring Model, as well as the multiple-factor generalized Arrhenius Model, Eyring Model, Pecking Model, etc. [1]. These models have been used to predict the creep life [26], fatigue life [27], and storage life [28] of T300 fiber-reinforced composites. Olesja [2] et al. summarized the usage conditions of various models, which were related to the environmental factors, performance degradation patterns, and failure mechanisms of materials. It is generally required that the failure mechanism does not change throughout the entire experimental process. The other model is a numerical simulation model, which fits equations based on data trends and has no physical meaning. This type of model has a relatively high degree of fit, but their application conditions are often limited.

At present, there is more research on performance changes and life predictions in hygrothermal environments for T300 fiber-reinforced polymer composites. However, research on T700 fiber-reinforced composites is few. Wang [29] investigated the hygrothermal behavior of T700 and T300 BMI and found that the tensile/compressive strength of two composites decreased in humid and hot environments, while the glass transition temperature became lower with the rate of water absorption. YU [30] found that, for T700 and T300 carbon fiber-strengthened epoxy resin composites aged at 70 °C, RH85% for 70 days, the longitudinal and transverse compressive strength and shear strength slightly reduced. The property retention rate of T700 carbon fiber-reinforced composites was slightly better. The surfaces of T300 fiber and T700 fiber exhibit significantly different characteristics, with T300 appearing as bark and having distinct grooves of varying depths. On the other hand, T700 presents a smooth surface [31]. This may lead to different resistances of T300 and T700 to humidity and heat.

In addition, the hygrothermal aging time of T700 polymer composites is relatively short, the long-term durability in humid and hot environments has not been fully reflected, and the storage life of T700 polymer composites is not focused. Current prediction models are mainly single-factor models and cannot simultaneously investigate the effects of temperature and humidity on composite properties.

In this article, the property degradation and hygrothermal failure mechanism of T700 fiber-reinforced polymer composites were investigated by long-term hygrothermal aging tests. A dual factorial life prediction model of temperature and humidity was established on the basis of property degradation and failure mechanism analysis. The natural storage life of T700 fiber-reinforced polymer composite in Guangzhou, China, where the temperature and humidity are high, was prediction.

## 2. Experimental Specimens and Experimental Methods

### 2.1. Specimens

**Figure 1:**
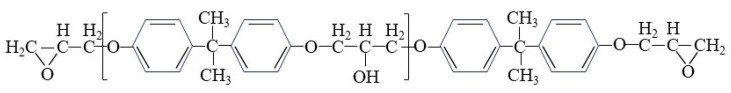
Schematic diagram of epoxy resin structure.

First, 200 mm × 200 mm carbon fiber-reinforced polymer matrix composite unidirectional laminates were prepared using T700 grade fibers with a fiber volume content of 60%. The polymer matrix was epoxy resin with an epoxy value of 85 eq/100 g, and a schematic diagram is shown in Figure 1. Composite laminates were made by wet winding method. The winding angle was 0°. Curing pressure was 0.6 MPa and curing process was 130 °C/4 h + 160 °C/2 h. The size of composite laminates was 300 mm × 300 mm.

Before hygrothermal aging and natural storage, composite laminate edges were sealed with HM108 sealant. After hygrothermal aging, laminates were machined by the precision cutting machine. In order to eliminate the edge effect of defects and moisture absorption, the laminates edges were removed by 25 mm.

The size of the moisture absorption specimen was 50 mm × 50 mm × 2 mm and the size of the interlayer shear specimen was 20 mm × 10 mm × 2 mm, which met the specimen requirements in the standard HB7401 [32]. The length and width accuracy of all specimens was ±0.02 mm and the thickness accuracy was ±0.05 mm, which met the specimen requirements in the standard JC-773 [33].

The samples were divided into two parts. One part was used for the aging test, while the other part was used for natural storage tests in Guangzhou, China.

### 2.2. Hygrothermal Accelerated Aging Test

Hygrothermal accelerated aging tests were conducted according to standard HB7401 [32] by constant temperature and humidity testing equipment with a temperature accuracy of ±0.2 °C and a humidity accuracy of ±RH2%. The hygrothermal accelerated aging conditions were 70 °C, RH70%, 70 °C, RH85%, 85 °C, RH70%, and 85 °C, RH85%, respectively. The test period was 360 days.

The masses of moisture absorption specimens were measured using a precision analytical balance with an accuracy of 0.0001 g. For the first four days, the specimen masses were measured every day, and then measured every three days. After reaching an effective moisture equilibrium, the masses of the specimens were measured every 10 days.

The moisture content ∆M was calculated using the following formula:

(1) $$∆M=\frac{W_{i}-W_{0}}{W_{0}}\times 100\%,$$

where

$W_{i}$—current specimen mass, g;

$W_{0}$—initial specimen mass, g.

When the moisture content change in the specimens is less than 0.02% for two consecutive times within 7 ± 0.5 days, it is considered that the specimens have reached an effective moisture equilibrium.

### 2.3. Interlayer Shear Test

**Figure 2:**
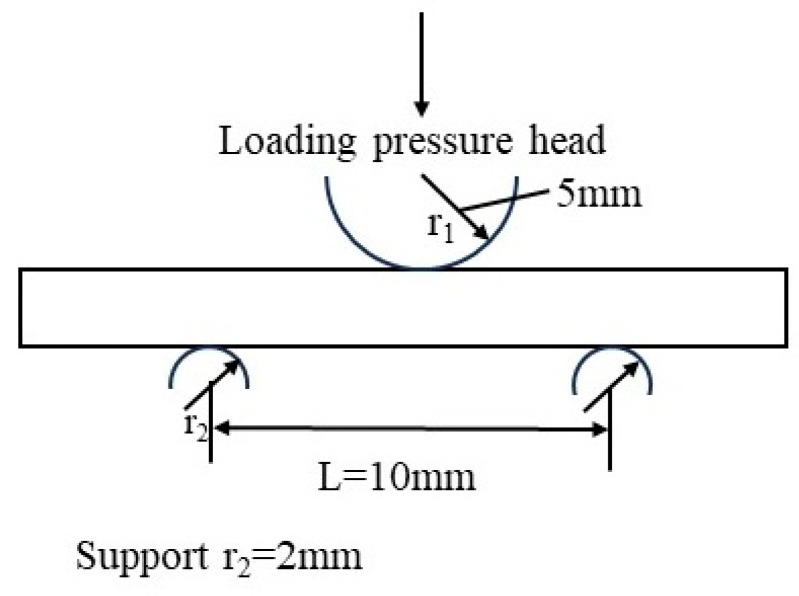
Schematic diagram of interlayer shear test.

After the moisture absorption test for 30 days, 60 days, 120 days, 180 days, 240 days, 300 days, and 360 days, 5 specimens were taken out and subjected to interlayer shear tests according to the standard JC/T773. A schematic diagram of the interlayer shear test is shown in Figure 2. The hardness of the loading head was 60–62 HRC, and there should be no burrs on the loading head surface. During the experiment, the loading head was equidistant from the supports on both sides, with an accuracy of ±0.3 mm. The movement speed of the loading head was 1 mm/min. Loading continued until the load dropped by 30% and the test was stopped. The interlayer shear strength was calculated by the following formula:

(2) $$\tau =0.75\times \frac{F}{bh},$$

where

τ—interlayer shear strength, MPa;

*F*—the maximum load, N;

b—specimen width, mm;

h—specimen thickness, mm.

### 2.4. Mechanism Analysis of Hygrothermal Accelerated Aging

To determine the changes in the type and content of characteristic functional groups of T700/epoxy resin before and after the hygrothermal accelerated aging test, infrared analysis was conducted on the unaged and aged specimens using the Spectrum 100 infrared spectrometer from PerkinElmer Company, Waltham, MA, USA.

The crystallization temperature and glass transition temperature of the resin matrix before and after hygrothermal accelerated aging were analyzed using the DSC25 differential scanning calorimeter from TA Company, New Castle, DE, USA, according to ASTM D3418 [34]. The test conditions were a nitrogen atmosphere, the heating rate was 10 °C/min, and the testing temperature range was −80 °C~300 °C.

The initial thermal decomposition temperatures of composite materials before and after aging were analyzed using the TGA2050 thermogravimetric analyzer from TA Company. The test conditions were a nitrogen atmosphere, the heating rate was 10 °C/min, and the testing temperature range was 30 °C~800 °C.

The specimens after interlaminar shear tests were embedded with epoxy resin in both vertical and parallel fiber directions. After curing at room temperature for 2 h, the specimens were ground using sandpaper with particle sizes of 200 #, 600 #, and 1200 #, then burnished using silicon dioxide polishing solution (particle size of 0.5 microns) and the metallographic samples were prepared. A thin layer of gold (about nm) was sprayed on the metallographic sample surfaces to increase the conductivity of the material using a KYKY-SBC-12 ion-sputtering instrument from KYKY Technology Company, Beijing, China. The conditions for spraying gold were as follows: voltage of 25 kV, vacuum degree of 6 Pa, and time of 45 s. The delamination, crack propagation path, interface changes between the fiber and resin, and resin change were analyzed using a field emission scanning electron microscope (FESEM).

### 2.5. Storage Life Prediction

On the basis of the failure mechanism analysis of hygrothermal accelerated aging, and the variation law analysis of interlayer shear strength, a temperature–humidity dual stress storage life prediction model was established.

Taking the Guangzhou region of China as an example, where high temperatures and humidity have a significant impact on the performance of composite materials (as shown in Table 1), the storage life of the composite under the actual storage conditions was calculated with the failure criterion of interlaminar shear strength degradation to 77% of the original value [35]. The reliability of the model was analyzed by comparing the prediction value and the actual measured value of specimens stored in Guangzhou for 2 years.

## 3. Results Analysis and Discussion

### 3.1. Moisture Content

**Figure 3:**
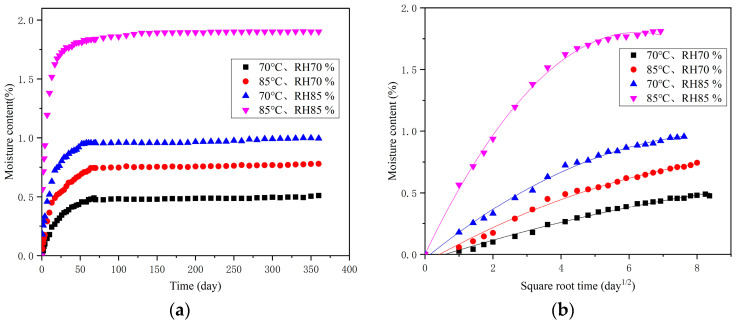
Moisture content curves with (**a**) the moisture content change with the accelerated aging time and (**b**) the initial stages of moisture content, which conform to Fick’s law.

Figure 3a shows that the moisture contents of the specimens increased, the times to reach effective moisture equilibrium decreased, and the moisture contents increased with increasing temperature and humidity under the accelerated conditions of 70 °C, RH70%; 70 °C, RH85%; 85 °C, RH70%; and 85 °C, RH85%. For example, the time to reach effective moisture equilibrium was 1320 h and the moisture content was 0.46% under the condition of 70 °C and RH70%, and the time to reach effective moisture equilibrium was 840 h and the moisture absorption was 1.77% under the conditions of 85 °C and RH85%. The moisture contents data under four temperature and humidity conditions were fitted nonlinearly. It was found that the moisture content changes meet Fick’s diffusion law before reaching effective moisture equilibrium, as shown in Figure 3b. The fitting correlation coefficients R2 were all higher than 0.988, showing a higher fitting degree. After reaching the effective moisture equilibrium, the moisture contents of the specimens changed slightly and showed a linear trend.

### 3.2. Interlayer Shear Strength under Different Hygrothermal Conditions

**Figure 4:**
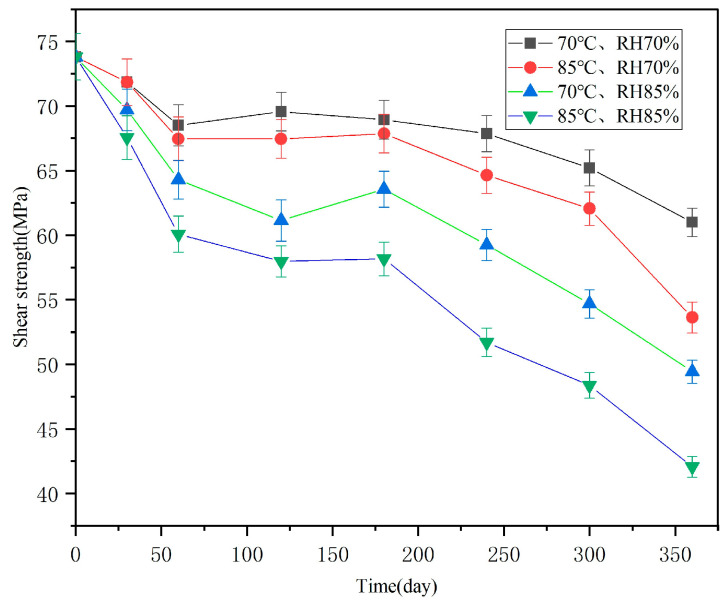
Interlaminar shear strength changes with accelerated aging time under different hygrothermal conditions.

The changes in interlayer shear strength with hygrothermal accelerated aging time are shown in Figure 4. It can be seen that, with increasing aging time, the interlaminar shear strengths showed a decreasing trend, and that the higher the temperature and humidity, the faster the interlaminar shear strengths declined. The data dispersions also showed an overall decreasing trend. For example, the average value of original interlaminar shear strength was 73.8 MPa, with a standard deviation of 1.8; after hygrothermal accelerated aging for 360 days under 85 °C and 85% conditions, the interlaminar shear strength average value was 42.1 MPA (decreased by 43%), with a standard deviation of 0.8. Obviously, the influence of moisture absorption on interlayer shear strength gradually increased, relatively reducing the influence of differences in sample preparation.

The interlayer shear strengths of specimens stored in Guangzhou for 2 years are shown in Table 2. The average value was 68.6 MPa; compared to the original performance of 73.8 MPa, this decreased by 7%.

### 3.3. Failure Mechanism of Hygrothermal Accelerated Aging

#### 3.3.1. Fourier Transform Infrared Analysis

**Figure 5:**
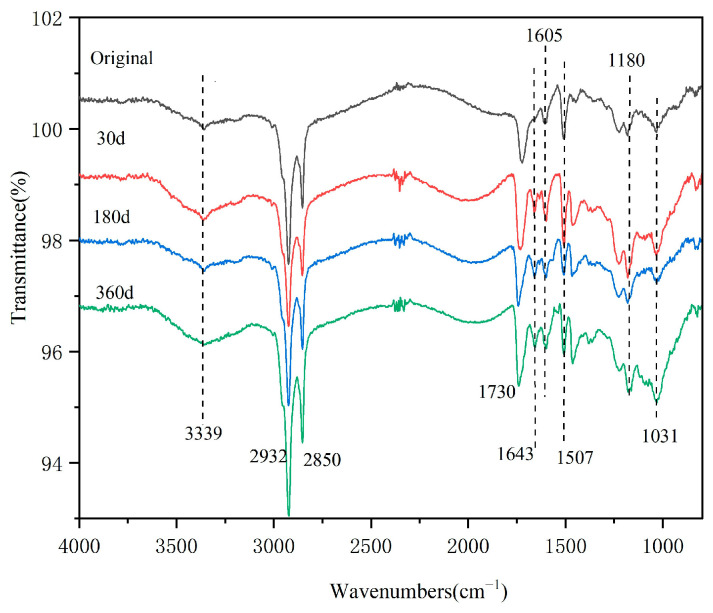
Infrared spectra of the specimens before and after hygrothermal accelerated aging.

Figure 5 shows the infrared spectra of the T700/epoxy composites before and after hygrothermal accelerated aging. The peak at 3339 cm^−1^ is the stretching vibration absorption peak of −OH. The peaks at 2932 cm^−1^ and 2850 cm^−1^ are the stretching vibration absorption peaks of saturated C-H.

The peak at 1730 cm^−1^ is the stretching vibration peak of C=O in the imine ring; at 1605 cm^−1^ and 1507 cm^−1^, there are stretching vibration peaks of the benzene ring skeleton C=C. The C-O stretching vibration peak is located at 1180 cm^−1^; 1031 cm^−1^ is the stretching vibration peak of the ether bond C-O-C.

After hygrothermal accelerated aging for 30 days to 360 days, the trend of infrared peak changes is the same. Only a small absorption peak is formed at 1643 cm^−1^, which is the stretching vibration absorption peak of the decomposition product–benzene ring produced by the slight hydrolysis of the resin [6]. This indicates that the resin slightly hydrolyzes after hygrothermal aging. The degree of resin hydrolysis does not increase when prolongating the aging time.

#### 3.3.2. DSC Analysis of Resin Matrix

**Figure 6:**
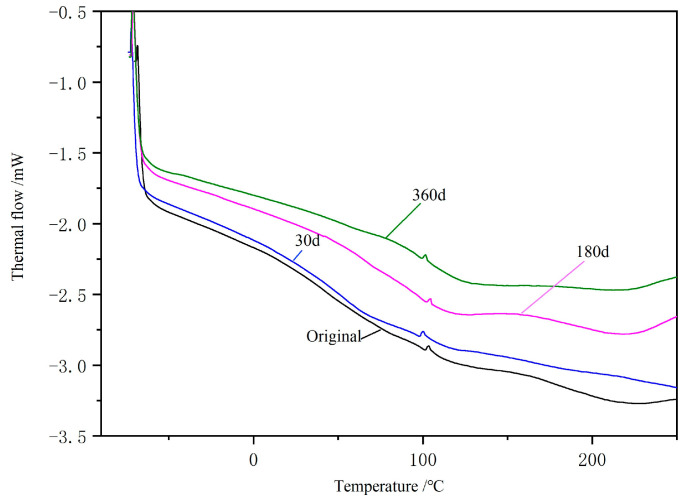
DSC results of resin matrix aged at 85 °C and RH85% for different times.

Figure 6 shows the DSC test curves of the resin matrix aged under 85 °C and RH85% conditions for different times. The glass transition temperature of epoxy resin is about 120 °C. An exothermic peak appears around 102 °C, which is a secondary reaction between the amino groups in the epoxy resin and curing agent.

It can be seen that there is no significant difference in the DSC curves between the original specimen and the specimens aged for different times. During the aging process, there was no significant chemical structural change in the matrix resin.

#### 3.3.3. TGA Results of Composites

**Figure 7:**
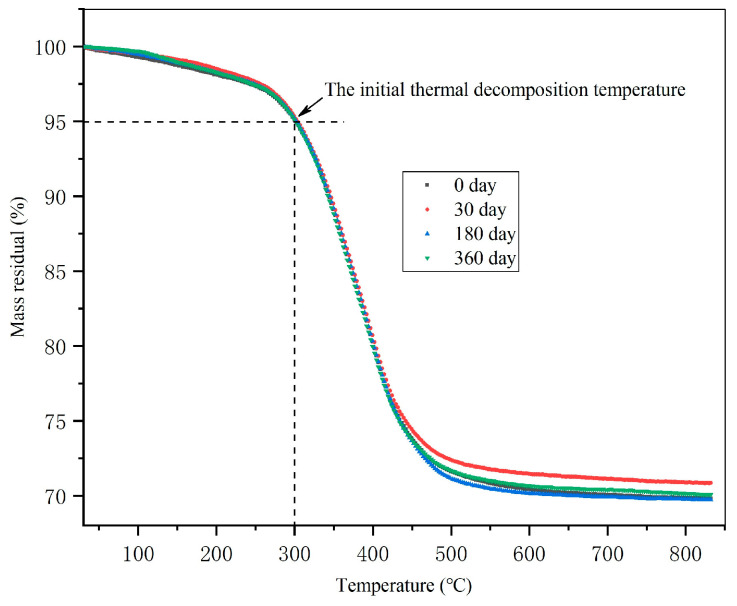
TGA results of resin matrix aged at 85 °C and RH85% for different times.

The TGA results of composites are shown in Figure 7. The results show that the initial thermal decomposition temperature of composite materials is about 300 °C. There was no significant change in the initial thermal decomposition temperature of the composite material before and after aging. There is a slight difference in the remaining mass of composites aged for different times, which is related to the different resin and fiber contents of the test samples. The TGA results further demonstrate that there is no significant chemical change in the resin matrix.

#### 3.3.4. Interlaminar Cracks and Interface Analysis

**Figure 8:**
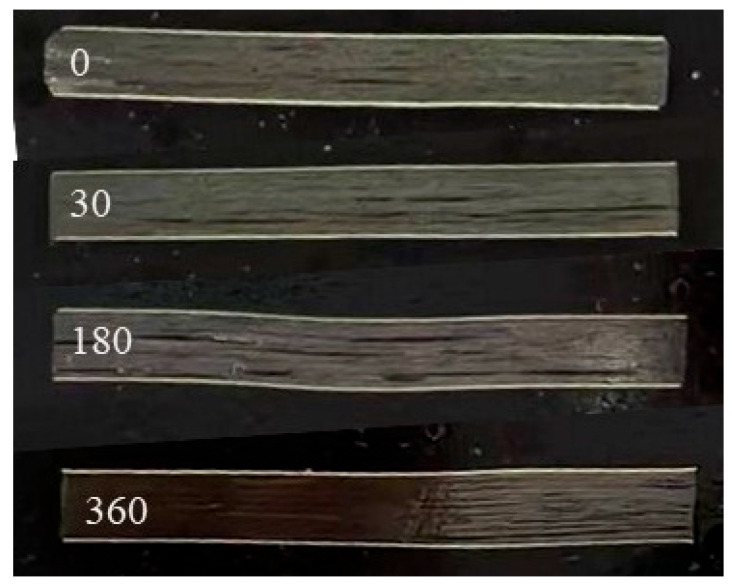
Interlayer cracks before and after hygrothermal accelerated aging.

Figure 8 shows that the number of delamination cracks observed from the specimen side increased with the hygrothermal accelerated aging time. Hygrothermal environments weaken the interface between the fiber and matrix, which is the main reason for the decrease in its shear strength.

**Figure 9:**
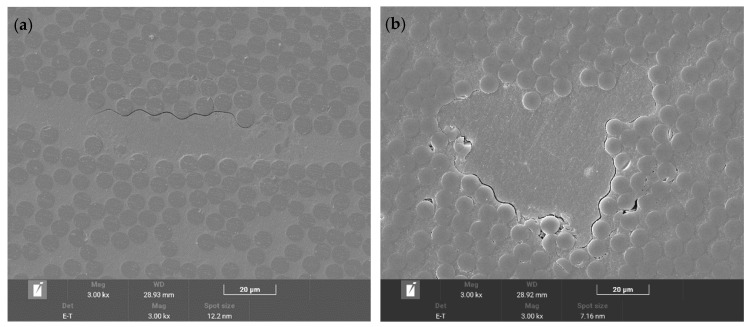

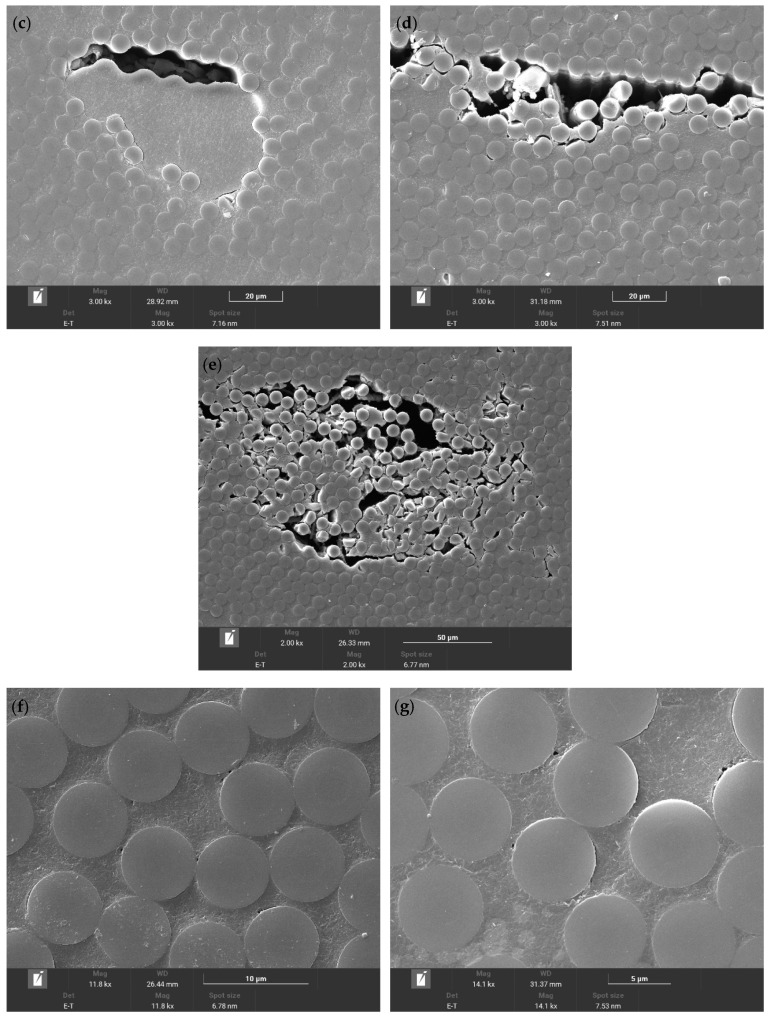
Interlaminar cracks and interface analysis of interlaminar shear specimens with (**a**) original; (**b**) hygrothermal accelerated aging for 30 days; (**c**,**d**) hygrothermal accelerated aging for 180 days; (**e**–**g**) hygrothermal accelerated aging for 360 days.

According to the FESEM analysis, the interlayer cracking of the unaged specimen was mainly located at the interface between fiber bundles and rich resin, which is a typical brittle cracking. There was no obvious cracking inside the matrix and fiber bundles, and the interface between the fibers and the matrix was well bonded in the non-rich resin area, as shown in Figure 9a. The uneven distribution of resin is also one of the reasons for the interlaminar shear strength dispersion. After hygrothermal accelerated aging for 30 days, cracks still mainly occurred at the interface between rich resin and fiber bundles, but the number and length of cracks increased significantly, as shown in Figure 9b. After hygrothermal accelerated aging for 180 days, cracks appeared at the interface between the resin and fibers, as well as within the fiber bundles, and the interface between all resin and fibers significantly weakened, as shown in Figure 9c,d.

The composites aged for 360 days showed more obvious cracking within the fiber bundles, as shown in Figure 9e, and pores caused by moisture absorption appeared at the interface between the fiber and matrix. There were no obvious pore characteristics inside the resin, as shown in Figure 9f,g.

#### 3.3.5. Fracture Morphology Analysis

**Figure 10:**
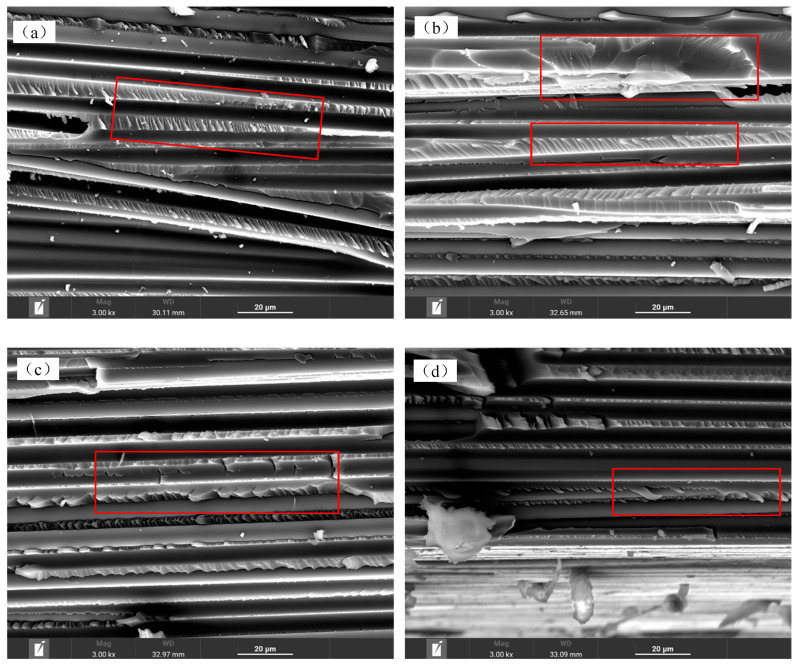
Fracture characteristics of interlaminar shear specimens with (**a**) original; (**b**) hygrothermal accelerated aging for 30 days; (**c**) hygrothermal accelerated aging for 180 days; (**d**) hygrothermal accelerated aging for 360 days.

The cracks were artificially opened, and the microscopic characteristics of the fracture surface were observed by FESEM. For the T700/epoxy composites without moisture and after hygrothermal accelerated aging for 30 days, the resin fracture characteristics were tear characteristics, which are typical brittle cracking, and the interfaces between the resin and fibers were well bonded, as shown in the red box in Figure 10a,b. With increasing hygrothermal accelerated aging time, resins were significantly elongated, which is typical ductile fractures. At the same time, adhesive resins on the fiber surface decreased the fiber surfaces became smooth, and the interface bond between the fibers and the resin matrix weakened, as shown in the red box in Figure 10c,d. And the resin fracture characteristics gradually became ductile with increasing aging time, which indicates that hygrothermal aging causes plasticization of the resin matrix.

From the above test results, it can be seen that the infrared and DSC tests of the resin matrix indicated that the resin matrix underwent slight hydrolysis after hygrothermal accelerated aging, but the degree of hydrolysis did not increase with the increase in hygrothermal accelerated aging time. The glass transition temperature of the resin matrix did not change significantly before and after hygrothermal aging. The chemical changes in the resin were not obvious. The fracture surfaces of the cracks showed that, with the increase in hygrothermal aging time, the resin matrix cracking gradually transitioned from brittle to plastic, and the resin matrix underwent plasticization.

An observation of the cross-section of the interlayer shear specimens revealed that cracks only appeared at the interface between the rich resin and fiber bundles in the non-aged and early hygrothermal accelerated aged specimens. With increasing hygrothermal accelerated aging time, cracks also appeared inside the fiber bundles, and pores caused by moisture absorption occurred at the interface between the fibers and the matrix. The main reasons for the decrease in the interlaminar shear strength were the gradual plasticization of resin moisture and the weakening of the interface between fibers and resin. The failure mechanism of the interlaminar shear did not obviously change throughout the whole hygrothermal accelerated aging process.

### 3.4. Storage Life Estimation

The life estimation model is based on the analysis of property degradation and the failure mechanism. If the property degradation law or failure mechanism of the interlaminar shear change during the hygrothermal accelerated aging test, different life prediction models would be used at different change stages. In this article, the degradation mechanism of the resin matrix remained unchanged during the whole hygrothermal accelerated aging test. Therefore, only one life prediction model was used throughout the whole hygrothermal aging process.

In general, the mechanical property degradation of polymer composites experiencing long-term hygrothermal effects decreases exponentially, which can be expressed by the following equation:

(3) $$y_{t}=y_{0}exp\left(-kt\right),$$

where:=

$y_{t}$—the mechanical property of composite materials at time *t*;

*y*_0_—the original mechanical property;

*k*—material property degradation rate;

*t*—hygrothermal accelerated aging time.

Under different hygrothermal conditions, the reaction rate of composite materials was different, and the time used for the performance to decrease to the same value was different.

**Figure 11:**
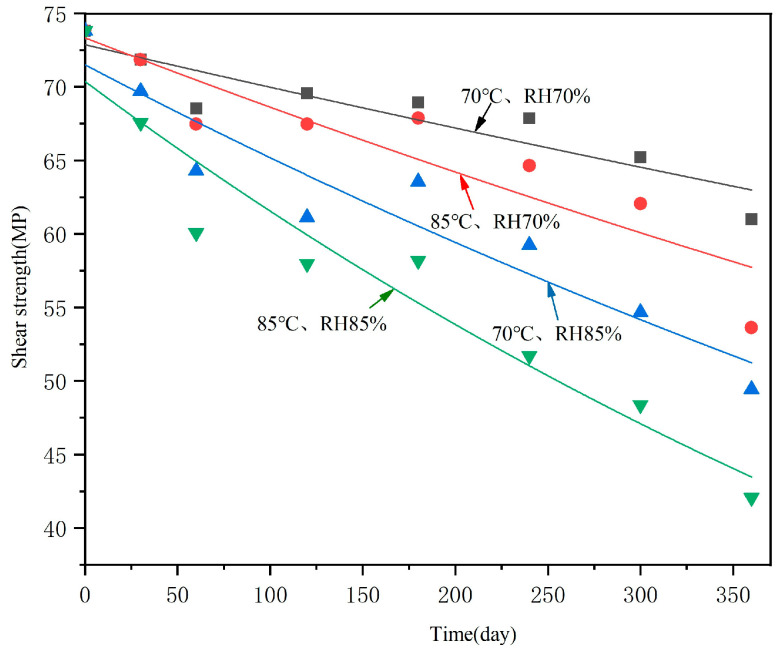
Regression curve of interlaminar shear strength with hygrothermal accelerated aging time.

The interlayer shear strengths of T700/epoxy, aged under the conditions of 70 °C, RH70%; 70 °C, RH85%; 85 °C, RH70%; and 85 °C, RH85%, were fitted using Origin software 2018. The degradation equations were obtained, as shown in Equations (4)–(7) and Figure 11.

(4) $$70\;{}^{\circ}C,RH70\%:y=72.86142e-0.0004t\left(\mathrm{fitting degree}\;R2=0.84\right),$$

(5) $$85\;{}^{\circ}C,RH70\%:y=73.33141e-0.00066t\left(\mathrm{fitting degree}\;R2=0.84\right),$$

(6) $$70\;{}^{\circ}C,RH85\%:y=71.49943e-0.00092t\left(\mathrm{fitting degree}\;R2=0.90\right),$$

(7) $$85\;{}^{\circ}C,RH85\%:y=70.36816e-0.00134t\left(\mathrm{fitting degree}\;R2=0.93\right),$$

The fitting degrees R2 of the above four equations are, respectively, 0.84, 0.84, 0.90, and 0.93.

The interlaminar shear strength degradation rates k of T700/epoxy resin under different hygrothermal accelerated aging conditions were obtained from the four equations above, as shown in Table 3.

Due to the accelerated test temperature being lower than the glass transition temperature of the matrix (90 °C), and there was no significant change in the glass transition temperature during the accelerated tests; the relationship between the interlaminar shear strength degradation rate of composite materials under two stress levels for humidity and temperature can be represented by the generalized Eyring’s Model [2]:

(8) $$k(T,H)=\frac{A}{T}e^{-\frac{Ea}{KT}}e^{H(C+\frac{D}{KT})}$$

where

A, C, and D are undetermined constants;

*Ea*—activation energy;

K—Boltzmann constant, 8.61738 × 10^−5^;

*T*—absolute temperature, K;

*H*—relative humidity, %.

For the convenience of calculation, Equation (8) is converted to a logarithmic form:

(9) ln(kT)=lnA+Ea/(KT)+CH+DH/(KT)

Let *a* = ln*A*, *b* = *Ea/K*, *c* = *C*, and *d* = *D/K*; Formula (9) can be simplified as

(10) ln(kT)=a+b/T+cH+dH/T

where

*a*, *b*, *c*, and *d* are the undetermined constants.

Substituting the reaction rates *k* at the conditions of 70 °C, RH70%; 85 °C, RH70%; 70 °C, RH85%; and 85 °C, RH85% (in Table 3) into Equation (10), the following equations can be obtained:

(11) −1.986316=a+0.0029155b+0.70c+0.002041d

(12) −1.442738=a+0.0027933b+0.70c+0.001955d

(13) −1.153406=a+0.0029155b+0.85c+0.002478d

(14) −0.734553=a+0.0027933b+0.85c+0.002374d

Four undetermined coefficients, *a*, *b*, *c*, and *d*, are obtained by solving Equations (11) to (14); thus, a two-factor reaction rate model regarding temperature and humidity for T700/epoxy resin is obtained:

(15) ln(kT)=20.9916−9214.6538/T−14.2922H+6806.8297H/T

Then, the interlaminar shear strength degradation rate at different temperatures and humidity levels k can be calculated from the following equation:

(16) k=exp ⁡(20.9916−9214.6538/T−14.2922H+6806.8297H/T)/T

The degradation rate under storage conditions of each season in Guangzhou, China can be obtained, as shown in Table 4.

The durability prediction methodology is based on the time shift concept—the ratio of the time which the performance composites take to decrease to a certain value at different humidities and temperatures [4].

According to Equation (3), the time required for the performance degradation to y_1_ of composite materials can be expressed as

$$t=ln{(y}_{1}/y_{0})/(-k)$$

Thus, the time shift factor (*TSF*) can be calculated by the following equation:

(17) $$TSF=\frac{t_{1}}{t_{2}}=\frac{ln{(y}_{1}/y_{0})/(-k_{1})}{ln{(y}_{1}/y_{0})/(-k_{2})}=\frac{k_{2}}{k_{1}}$$

Thus, it can be concluded that the *TSF* of the laboratory at 85 °C, RH85% under the storage conditions of spring, summer, autumn, and winter in Guangzhou, China, are 39.4118, 11.0744, 47.8571, and 89.3333, respectively. In other words, the entire spring, summer, autumn, and winter storage period in Guangzhou, China is equivalent to 2.3153 days, 8.2397 days, 1.9067 days, and 1.0214 days stored under laboratory conditions of 85 °C and RH85%. Assuming that the interlaminar shear strength degradation is a cumulative damage process during the whole storage process, the storage time of T700/epoxy resin composite in Guangzhou, China for one year is equivalent to 13.4831 days under the laboratory conditions of 85 °C and RH85%. Thus, the equivalent relationship between actual storage and accelerated testing is obtained.

According to the safety factor guidelines of the Federal Aviation Administration for fiber-reinforced resin based composite laminates [35], the failure criterion is that the strength of composite materials decreases to 77% of the original value, meaning that

(18) $$F_{failure}=0.77F_{unaged}\;$$

when $\frac{y_{t}}{y_{0}}=0.77$, the storage time of the T700/resin matrix composite at 85 °C and RH85% is 195.0483 days, and the storage life in Guangzhou, China is 14.4661 years.

The interlayer shear strength of T700/epoxy resin measured after 2 years of actual storage is 68.6 MPa, as shown in Table 2. Based on the equivalent relationship between actual storage and laboratory storage at 85 °C and RH85%, the interlaminar shear strength calculated for two years storage in Guangzhou, China is 67.87 MPa. The calculated values are close to the measured values, and the model is effective. The model still needs to be validated with more actual stored data.

## 4. Conclusions

The durability of carbon fiber-reinforced polymer composites was investigated by hygrothermal accelerated aging tests for 360 days and a natural storage test. Based on the analysis of property changes and the hygrothermal aging mechanism, a dual factor storage life prediction model was established. The main conclusions obtained are as follows:

(1) The order of moisture absorption rates, moisture absorption contents, and the severity effect order on the interlaminar shear strength is RH85%; 85 °C > 70 °C, RH85% > 85 °C, RH70% > 70 °C, RH70%. The times to achieve effective moisture absorption balance were opposite to this.
(2) The moisture absorption rate satisfies Fick’s law before the effective moisture absorption balance, then shows a linear trend. The interlayer shear strength still decreases exponentially with aging time, which is mainly caused by the resin plasticization and interface weakening.
(3) With the increase in hygrothermal accelerated aging time, the resin matrix fracture gradually changes from a brittle fracture to a plastic fracture. The main reasons for the interlayer shear strength decreasing are resin moisture plasticization and interface weakening.
(4) A dual stress storage life prediction model and the equivalent relationship between natural storage and hygrothermal acceleration were established. According to the model, based on the failure criterion of the interlayer shear strength decreasing to 77% of the initial value, the storage life of T700/epoxy resin in Guangzhou, China was calculated to be 14.4661 years. The effectiveness of the model was verified by actual storage data for 2 years.

## Figures and Tables

**Table 1:** Temperature and humidity conditions of actual storage in Guangzhou, China.

Storage Time	Average Temperature and Humidity
Spring (From March to May)	21 °C ± 0.5 °C, RH65% ± RH 2%
Summer (From June to August)	29 °C ± 0.5 °C, RH 75% ± RH 2%
Autumn (From September to November)	25 °C ± 0.5 °C, RH60% ± RH 2%
Winter (From December to February)	18 °C ± 0.5 °C, RH58% ± RH 2%

**Table 2:** Interlayer shear strength of specimens stored in Guangzhou for two years.

Interlayer Shear Strength (MPa)	Average Value (MPa)	Coefficient of Variation (%)
68.3	68.6	1.6
69.0		
70.0		
67.0		
68.5		

**Table 3:** Interlaminar shear strength degradation rate *k* under different hygrothermal accelerated aging conditions.

Hygrothermal Accelerated Aging Conditions	Degradation Rate *k*
70 °C, RH70%	0.0004
85 °C, RH70%	0.00066
70 °C, RH85%	0.00092
85 °C, RH85%	0.00134

**Table 4:** Degradation rate of interlayer shear strength stored in Guangzhou, China.

Storage Conditions	Degradation Rate k
Spring (From March to May)	0.000034
Summer (From June to August)	0.000121
Autumn (From September to November)	0.000028
Winter (From December to February)	0.000015

## Data Availability

Data are contained within the article.
